# Influence of calcareous nodules content on scaling effect in shear strength of cohesive soil containing calcareous nodules

**DOI:** 10.1038/s41598-021-04333-1

**Published:** 2022-01-10

**Authors:** Chuan-yang Liang, Yue-dong Wu, Jian Liu, Lei Zhang, Lai-he Lin, Da-shuo Chen

**Affiliations:** 1 Key Laboratory of Ministry of Education for Geomechanics and Embankment Engineering, Hohai University, Nanjing, 210024 China; 2 Geotechnical Engineering Research Center of Jiangsu Province, Nanjing, 210024 China; 3 Fujian Provincial Institute of Architectural Design and Research Co. Ltd., Fuzhou, 350000 China

**Keywords:** Civil engineering, Engineering

## Abstract

The difference in the shear strength and other characteristics of the cohesive soil containing calcareous nodules (CSCN) between samples with large size and corresponding scaling size, which is called scaling effect, is significantly affected by its calcareous nodule content (CNC) of the gradation composition. However, current researches rarely reveal the influence of the CNC on the scaling effect in shear strength of samples. In this study, how and why the CNC affects the scaling effect in shear strength were explored. Then a method to reduce the scaling effect based on the reason for influence was proposed. Results show that the correlation between the scaling effect in shear strength and the CNC presents a step curve. This is attributed to that it is easier to form a skeleton effect in samples with scaling size for the same CNC. Considering the skeleton effect, a calculation model for the shear strength parameters of CSCN samples with large size is proposed to reduce the scaling effect. This paper demonstrates that the proposed calculation model provides an access to obtain calculated shear strength parameters of CSCN samples with large size by using measured results of samples with corresponding scaling size.

## Introduction

Cohesive soil containing calcareous nodules (CSCN) is a special soil-rock mixture^1,2^, which is composed of soil and calcareous nodules^3,4^. In lab direct shear tests, since the particle size of calcareous nodules is generally between 1 and 80 mm^3^, a large-scale direct shear apparatus of which the ring with 300 mm in diameter and 150 mm in height^5^ is preferred to be used for CSCN samples. These CSCN samples with the same size as that of the above ring are called samples with large size^6^. During the process of the sample preparation, the time of the preparation for a reconstituted sample with large size is long and the cost of that is high^7^, which incurs the huge economic and time costs to carry out multiple groups of tests at the same time for some research institutes. In order to shorten the sample preparation time and cut down the test cost, samples with the diameter of 61.8 mm and the height of 20 mm, which are called samples with scaling size, are considered to replace samples with large size to study the shear properties of the CSCN^8^. Furthermore, calcareous nodules with the size of more than 5 mm (oversize calcareous nodules) in samples with scaling size should be processed to meet the requirement that the maximum size of soil particles in samples is 1/5 ~ 1/6 of the sample diameter^6^.

At present, the “oversize calcareous nodules” processing methods that are commonly adopted in lab tests, including the oversize elimination, equal quantity substitution and similar gradation methods, inevitably affect the macroscopic deformation and failure mechanisms of CSCN samples^9^. For example, the oversize elimination method that sieving oversize calcareous nodules from CSCN samples decreases the calcareous nodule content (CNC) to change the gradation composition of the sample^10^, decreasing the shear strength of CSCN samples with scaling size^11^. In other words, the difference of the gradation composition results in the difference in the shear strength and other characteristics of the CSCN between samples with large size and corresponding scaling size, which is called scaling effect^6,12^.

According to previous studies, the difference of gradation composition is mainly reflected in the difference of maximum particle size and content of grain group of calcareous nodules^13^. Many researches focus on the influence of the maximum particle size on the scaling effect of samples^14,15^. For example, the cohesion and internal friction angle^16^, maximum dry density^17^ of samples with scaling size which are prepared by the similar gradation method decrease with the decrease of the maximum particle size in gradation composition. On the other hand, many gradation equations based on the gradation composition have been proposed, which study the influence of gradation composition on the scaling effect by changing the equation fitting parameters^13,18,19^. However, these equations cannot directly reveal the influence law of grain group content of calcareous nodules, i.e., the CNC (total content of each grain group) on the scaling effect in soil strength. The current research on how and why the scaling effect in shear strength would be affected by the CNC is still superficial or even absent.

In this paper, a series of direct shear tests were carried out on CSCN samples with large size and corresponding scaling size with different CNCs, respectively. The influence of the CNC on the scaling effect in shear strength of CSCN samples was studied systematically. From an understanding of the influence, a calculation model for the cohesion and internal friction angle of CSCN samples was proposed to reduce the scaling effect. The proposed model was then verified with data obtained from the published dissertations.

## Methodology

### Test plans

Table 1 lists the test plan, where L and S represent the sample with large size and corresponding scaling size, respectively. In order to study the influence of the CNC on the scaling effect in shear strength of the CSCN, the direct shear tests of samples with large size (*Ф* 300 × *H* 270 mm) and corresponding scaling size (*Ф* 61.8 × *H* 20 mm) were carried out, respectively. Six groups of samples with 0%, 10%, 20%, 30%, 40% and 50% in CNC were set for each type of test, respectively. In addition, the particle breakage of calcareous nodules can be ignored at the small normal stress^9^. Accordingly, the maximum normal stress of 200 kPa was applied in this test to reduce the influence of the particle breakage of calcareous nodules on the test results.

**Table 1:** Test plans of the direct shear test.

Type	CNC (%)	Sample dimension	
		Diameter, *Ф* (mm)	Height, *H* (mm)
L	0	300	270
	10		
	20		
	30		
	40		
	50		
S	0	61.8	20
	10		
	20		
	30		
	40		
	50		

### Test apparatus

**Figure 1:**
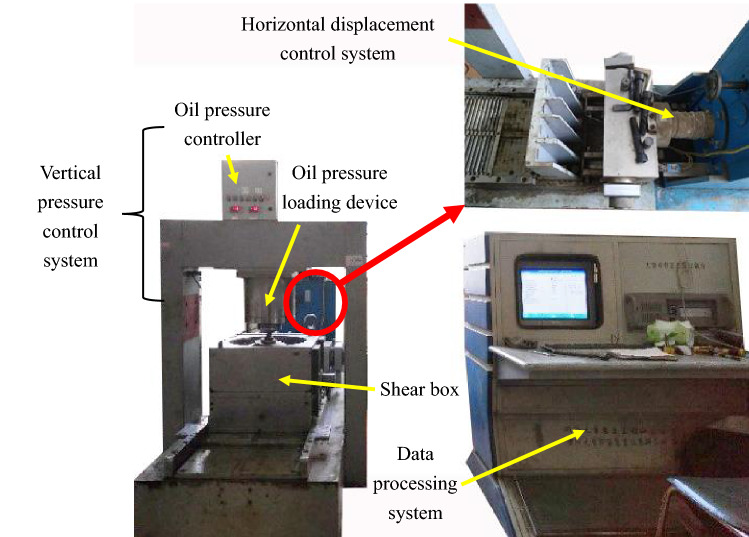
Photos of the large-scale direct shear apparatus.

Figure 1 shows photographs of the large-scale direct shear apparatus (SS-300) jointly developed by institute of geotechnical instruments of Sichuan University and Hohai University. The large-scale direct shear apparatus was mainly composed of a vertical pressure control system, a horizontal displacement control system, a shear box and a data processing system. The vertical pressure control system included an oil pressure controller and an oil pressure loading device. The horizontal displacement control system was used to control the rate at which the sample was shearing. The inner diameter of this shear box was 300 mm and its height was 270 mm, which was suitable for the sample with the maximum particle size of 60 mm. In the process of shearing, the data processing system can display the vertical normal stress, vertical strain, shear stress, horizontal displacement and their relationship in real time. Differently, the size of the shear box of the normal-scale direct shear apparatus was 61.8 mm in inner diameter and 20 mm in height, which was suitable for the sample with the maximum particle size of 2 mm.

### Test materials

**Figure 2:**
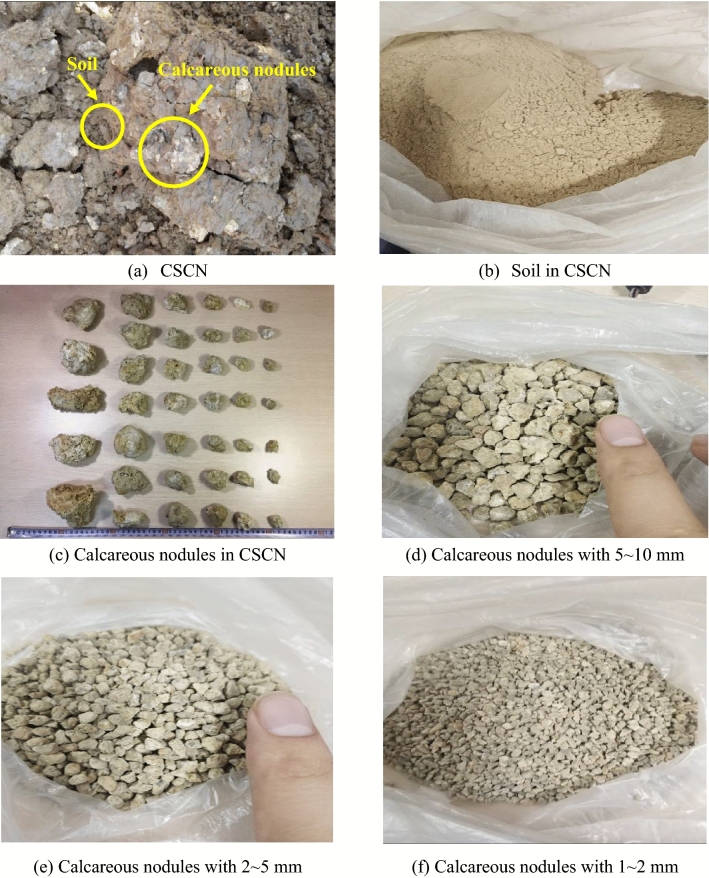
Typical constituents and structural characteristics of CSCN.

**Figure 3:**
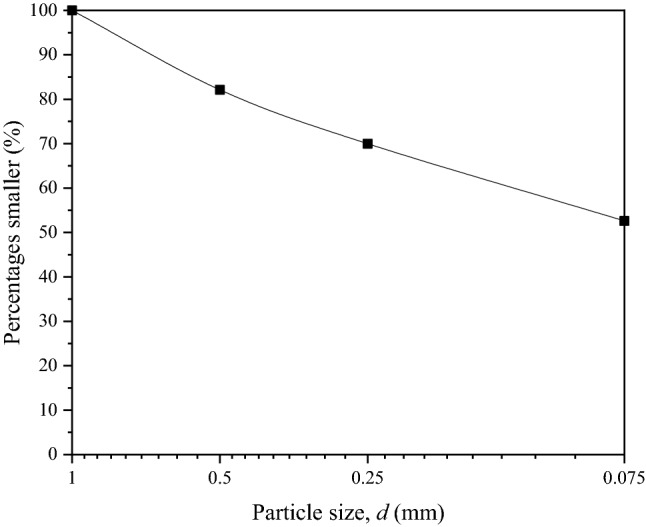
Gradation distributions of soil in the CSCN samples.

In this study, the CSCN was taken from Suqian, China and its typical constituents and structural characteristics are shown in Fig. 2. It can be seen that the CSCN is composed of soil and calcareous nodules with different particle sizes. The calcareous nodules accounted for around 20% with the particle size in range of 1–40 mm in natural CSCN. Figure 3 shows the gradation distributions of soil in the CSCN samples. It can be seen that more than 50% of soil by dry weight past the No. 200 (0.075 mm) sieve, which indicates that the soil should be classified as fine-grained soil. Basic indices which include plastic limit and liquid limit for the soil were determined for classification purposes. The physical parameters of the soil in CSCN are listed in Table 2. According to the Unified Soil Classification System (ASTM D2487-11), the fine-grained soil in test CSCN was classified as sandy fat clay (CH).

**Table 2:** Physical parameters of the soil in CSCN.

Dry density, *ρ*_d_ (g/cm^3^)	Water content, *w* (%)	Liquid limit, *w*_L_ (%)	Plastic limit, *w*_p_ (%)	Plasticity index, *I*_p_
1.75	17.90	51.02	25.20	26.0

### Sample preparation

**Figure 4:**
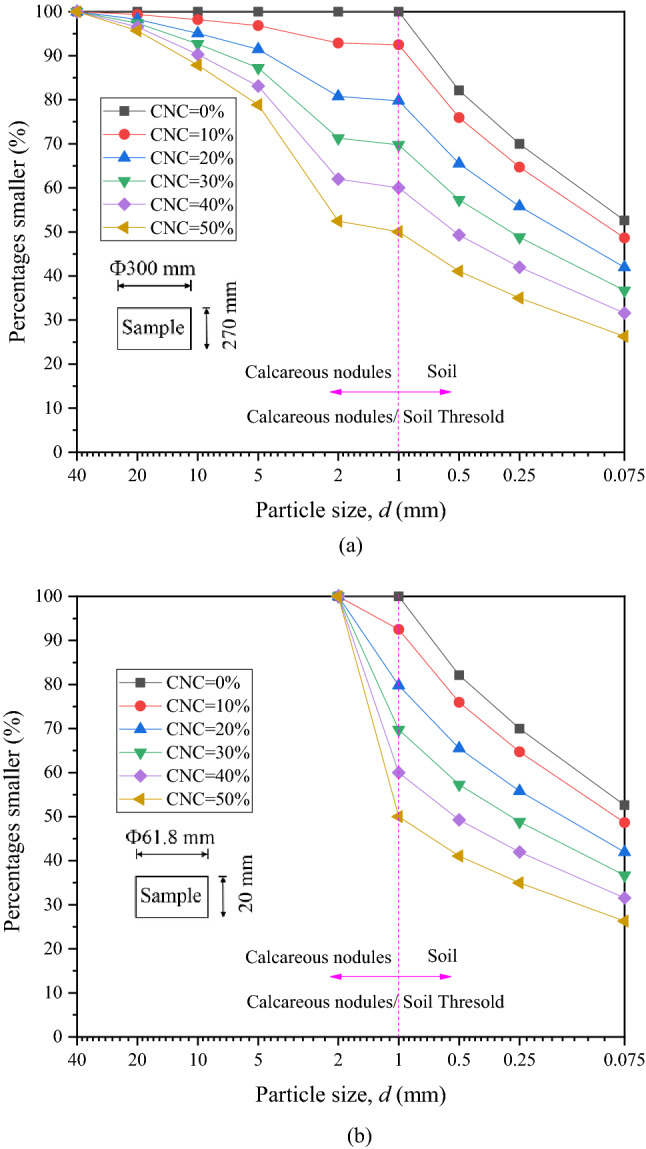
Gradation distributions of CSCN samples with (**a**) large size and (**b**) scaling size.

Samples with large size and corresponding scaling size were prepared from the natural CSCN. First, the calcareous nodules were isolated from the natural CSCN and sieved to obtain the grain group with 1–2 mm, 2–5 mm, 5–10 mm, 10–20 mm and more than 20 mm. A predetermined quantity of calcareous nodules was added to the soil and then mixed through a mixing machine to obtain different samples with CNC of 0%, 10%, 20%, 30%, 40% and 50%. The proportion of each grain group of calcareous nodules in each mixture was consistent with that in natural CSCN, as shown in Fig. 4a. The homogeneous mixture was transferred into rings in layers to control the compactness of 90% according to the compactness standard of urban road soil subgrade. All the samples were soaked under water and were then subjected to vacuum for 8 h to facilitate saturation. This vacuum time is generally regarded as sufficient to eliminate the impact of suction on consolidation^20^.

Differently, oversize calcareous nodules in samples with scaling size were first processed to within 2 mm applying the similar gradation mixed with equal mass substitution method, based on ASTM. This method can well control the content ratio of each grain group of calcareous nodules in samples with scaling size similar to that in samples with large size for the same CNC^13^. In order to control the same shear properties of soil, the particle size of soil in the CSCN was not scaled. Then the gradation distributions of samples with scaling size are shown in Fig. 4b.

Direct shear tests for CSCN samples were carried out using the large-scale (*Ф* 300 × *H* 270 mm) and normal-scale (*Ф* 61.8 × *H* 20 mm) direct shear apparatus, respectively, in accordance with ASTM D3080/D3080M-11. Normal load applied were 353 kg, 707 kg, 1060 kg and 1413 kg for samples with large size. Before shearing, samples were consolidated for 24 h under 50 kPa, 100 kPa, 150 kPa and 200 kPa of normal stress, respectively. Strain controlled test was the type that was carried out with the sample sets and the apparatus applied strain at the rate of 0.8 mm/min.

## Results and discussions

### Influence on scaling effect in shear strength

**Figure 5:**
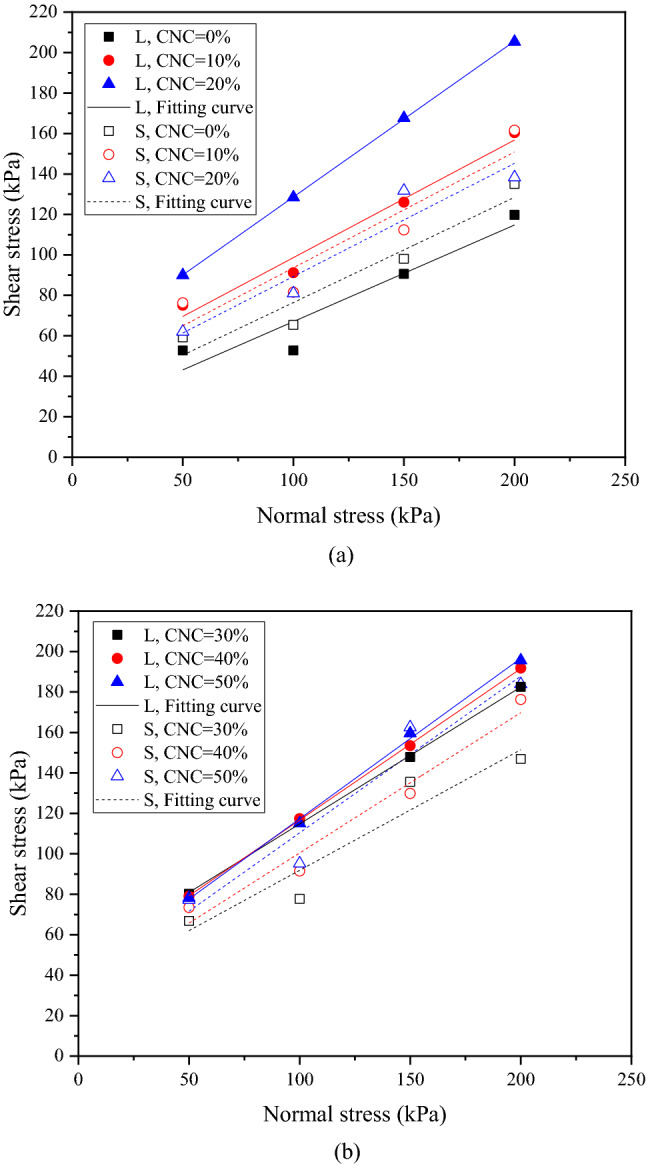
Shear stress versus normal stress plot of CSCN samples with large size and corresponding scaling size at the CNC of (**a**) 0%–20% and (**b**) 30%–50%.

Figure 5a and b show plots of shear strength due to the CNC of 0%–20% and 30%–50%, respectively, where L represents the sample with large size and S represents the sample with corresponding scaling size. For a certain sample size, the fitting curve of shear stress of samples with a higher CNC lies above that with a lower CNC. This behavior is attributed to the greater shear strength due to the increase of the CNC at a given normal stress. In addition, when the CNC of samples is same, the fitting curve of shear stress of samples with large size lies above that with corresponding scaling size. It means that there is a scaling effect in shear strength between samples with large size and corresponding scaling size. It is interesting to note that for a given normal stress, with the increase in the CNC, the distance of fitting curves between samples with large size and corresponding scaling size increases at the CNC of 0%–20%, as shown in Fig. 5a, while the distance decreases at the CNC of 30%–50%, as shown in Fig. 5b. This suggests that a greater degree of influence on the scaling effect in shear strength is caused by the CNC of 20%–30%.

Within the range of normal stress in the test, the relationship between shear strength and normal stress of samples with large size and corresponding scaling size fit the Mohr–Coulomb strength theory model^21^:

1 $$\tau_{{\text{f}}} = c + \sigma \cdot \tan \varphi$$

where: $\tau_{{\text{f}}}$ represents the shear stress of samples, kPa; $\sigma$ represents the normal stress, kPa; $c$ represents the cohesion, kPa; $\varphi$ represents the internal friction angle, °.

In order to explore the effect of the CNC on the scaling effect in shear strength quantitatively, two key parameters of typical plots of shear stress versus normal stress for each CSCN sample, cohesion and internal friction angle, were extracted to study their relationships with the CNC.

**Figure 6:**
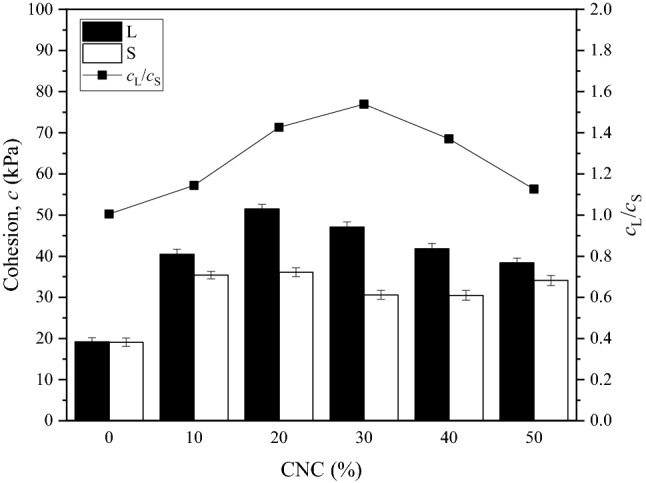
Relationship between the cohesion of CSCN samples and the CNC.

Figure 6 shows the relationship between the cohesion of CSCN samples and the CNC. For a certain sample size, a higher CNC yields a cohesion increasing first and then decreasing, which is consistent with previous studies^22^. For instance, with an increase in the CNC from 0 to 50%, the cohesion of samples with large size significantly increases from less than 20 kPa to more than 50 kPa then decreases to around 35 kPa, while that of samples with corresponding scaling size increases from less than 20 kPa to more than 35 kPa then decreases to around 30 kPa. It is interesting to note that the cohesion of samples with large size and corresponding scaling size reaches to the largest value at the CNC of around 20%, i.e., 51.5 kPa and 36.1 kPa, respectively, but difference of the cohesion is the largest at the CNC of around 30%. This behavior suggests that the influence of the CNC on the scaling effect in cohesion is slightly different from that on the cohesion of samples. Then the ratio (*c*_L_/*c*_N_) of the cohesion of samples with large size and corresponding scaling size for the same CNC is used to express the scaling effect in the cohesion. It is worth noting that, the ratio is around 1.0 when the CNC is 0%–10% and 50% but which rises to around 1.5 when the CNC is 20%–30%. It suggests that there is an inflection point in the correlation curve of the scaling effect in the cohesion and CNC, which presents a step curve. In other words, the cohesion of samples with large size decreases at the CNC of 20%–30% while that for samples with corresponding scaling size at the CNC of 10%–20%. It is attributed to the significant role of resistance to shear stress a low CNC played in samples with corresponding scaling size, which is equivalent to that of a high CNC in samples with large size.

**Figure 7:**
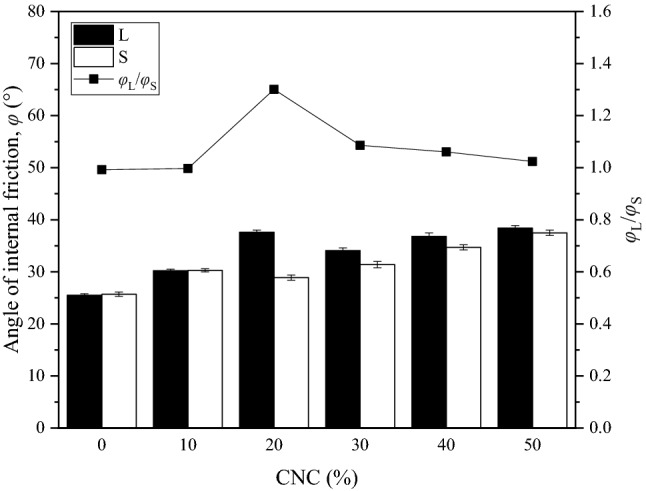
Relationship between the angle of internal friction of CSCN samples and the CNC.

Figure 7 shows the relationship between the internal friction angle of CSCN samples and the CNC. For a given sample size, the internal friction angle shows an increased tendency with the increase of the CNC. The increasing internal friction angle with the increasing CNC is due to the stronger bite force enhanced by calcareous nodules^23^. Similar to the development trend of the cohesion, the difference of the internal friction angle of samples with 20% in CNC is the largest, approximately 8.7º. This suggests that the scaling effect in the internal friction angle is insignificant for samples with a low (0%–10%) or high (more than 30%) CNC. Accordingly, at this time, it is applicable to adopt samples with scaling size to study the shear properties of the CSCN without considering the scaling effect. As expected, there is also an inflection point in the correlation curve of the scaling effect in the internal friction angle with CNC. To be specific, the ratio (*φ*_L_/*φ*_S_) of the internal friction angle of samples with large size and corresponding scaling size for the same CNC climbs to the largest, approximately 1.3 at the CNC of 20%. Hence, a great degree of bite force is associated with a high CNC in samples with large size while that with a low CNC in samples with corresponding scaling size.

### Reasons for influence on scaling effect

**Figure 8:**
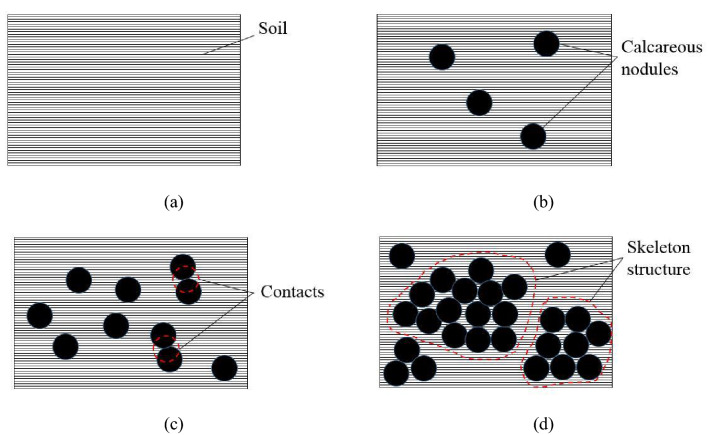
Schematic diagram of skeleton effect formation of calcareous nodules with CNC of (**a**) 0%, (**b**) 10%–20%, (**c**) 20%–30% and (**d**) 40%–50%.

Xu and Zhang^9^ pointed out that the primary reason for the influence of the CNC on the shear strength of samples was due to a skeleton effect formed by calcareous nodules. The skeleton effect here mainly refers to the influence of the framework formed by the contacts of calcareous nodules in the sample on the deformation and strength. A large number of calcareous nodules gather together, contact each other and transfer interaction, which will easily form a strong skeleton effect^24^. Figure 8 shows the schematic diagram of the skeleton effect formation of calcareous nodules. When the CNC is 0%–10%, the main component in CSCN samples is the fine soil, in which a small number of calcareous nodules suspend, as shown in Fig. 8a and b. The distance between calcareous nodules is large, making it difficult for calcareous nodules to interact with each other. At this time, the shear strength is mainly reflected by the cohesion and internal friction angle of the fine soil, which causes the small difference in the shear properties of the CSCN between samples with large size and corresponding scaling size. This suggests that a small amount of the CNC insignificantly affects the scaling effect in the macro-mechanics of CSCN samples. With the increase of the CNC, calcareous nodules are in close contact with each other and the fine soil fills in pores among calcareous nodules. The distance between calcareous nodules decreases and interactions occur gradually, which is expressed by the increase of contacts of calcareous nodules^22,25^, as shown in Fig. 8c and d. A great amount contacts of calcareous nodules form a strong skeleton structure in samples to resist the shear, resulting in the skeleton effect. This behavior supports a strong "pseudo cohesion"^26^ and bite force to increase the shear properties of samples, which is the common reflection of the interaction between calcareous nodules and soil^9,27^. When the CNC is more than a certain content, the skeleton structure in samples is almost completely built, which results in that the "pseudo cohesion" and bite force provided by calcareous nodules gradually reaches to a limit state^28^. Hence, the increase of CNC creates an insignificant difference in the macro-mechanics between samples with large size and corresponding scaling size.

The great difference of scaling effect in shear properties of samples is associated with the skeleton effect caused by soil structure in CSCN samples during different levels of CNC. The skeleton effect works effectively in samples with scaling size at the CNC of 10%–20% but it does not work in samples with large size until the CNC is 20%–30%. This behavior is attributed to the larger ratio of the particle size of calcareous nodules with the largest content in samples with scaling size to the corresponding sample diameter^29^. In samples with large size, the grain groups of calcareous nodules are widely distributed from 1 to 40 mm. In order to make the gradation curves with continuous gradation, there is a certain content of calcareous nodules in each grain group^19^. During the preparation for samples with corresponding scaling size, the grain group distribution range of calcareous nodules is reduced to between 1 and 2 mm. For the same CNC, more calcareous nodules with scaling particle size are arranged into the downsized grain group, which increases the content in this grain group. In other words, this behavior makes the content of grain group of 1–2 mm the largest, which affects significantly the soil structure. As the similar gradation composition, compared with that for samples with large size, the ratio of the particle size of calcareous nodules with the largest content in samples with corresponding scaling size to the sample diameter is larger. To be specific, for samples with scaling size, the content of calcareous nodules with 1–2 mm in particle size is the largest and the ratio of this particle size to the sample diameter is 1/60–1/30, as shown in Fig. 4b. While for samples with large size, the ratio of the particle size of 2–5 mm accounting for the largest content to the sample diameter is 1/150–1/60, as shown in Fig. 4a. Hence, it is easier for calcareous nodules in samples with scaling size to form a skeleton structure. During the consolidation stage, this skeleton structure can resist the compression stress, which results in a low consolidation degree of samples. Then, this causes the shear strength of samples with scaling size smaller than that of samples with large size, i.e., the scaling effect is increased easily during the process of shearing.

### Calculation model to reduce scaling effect

In order to reduce the scaling effect in shear strength, many researches study the calculation of the shear strength parameters of samples with large size by modifying that of samples with scaling size^14,15,30^. For example, Li et al.^6^ proposed a modification method for shear properties of samples with large size using the modifying coefficient, which is expressed by Eqs. (2)–(6).

2 $$R_{{\text{d}}} = \frac{{d_{{\text{m,field}}} }}{{d_{{\text{m,lab}}} }}$$

3 $$C_{\upvarphi } = a(R_{{\text{d}}} )^{T}$$

4 $$C_{{\text{c}}} = b(R_{{\text{d}}} )^{U}$$

5 $$\varphi_{{{\text{field}}}} = C_{\upvarphi } \cdot \varphi_{{{\text{lab}}}}$$

6 $$c_{{{\text{field}}}} = C_{{\text{c}}} \cdot c_{{{\text{lab}}}}$$

where $R_{{\text{d}}}$ represents the scaling coefficient of the particle size, $d_{{\text{m,field}}}$ represents the largest particle size in field, $d_{{\text{m,lab}}}$ represents the largest particle size in lab, $C_{\varphi }$ and $C_{{\text{c}}}$ represent the modifying coefficients of the shear properties, *a*, *T*, *b* and *U* represent the test constants of samples in lab, $\varphi_{{{\text{field}}}}$ and $c_{{{\text{field}}}}$ represent the shear properties of samples in field, $\varphi_{{{\text{lab}}}}$ and $c_{{{\text{lab}}}}$ represent the shear properties of samples in lab.

However, these calculation methods only consider the law of the difference in the shear properties between samples with large size and corresponding scaling size, which is the result caused by the scaling effect, ignoring the skeleton effect which is the reason for the scaling effect. To overcome this problem, a calculation model for shear strength parameters of CSCN samples considering the skeleton effect is investigated. First of all, from an understanding of the reason for the influence on the scaling effect in shear strength, the relationship between contacts of calcareous nodules which can express the skeleton effect and shear properties of samples is revealed. Shi et al.^31^ pointed out that the skeleton contacts can only transfer elastic interaction but cannot resist tension and rotation. Based on this, contacts of calcareous nodules can be obtained based on the following two assumptions. First, all the calcareous nodules are assumed to be rigid spheres with same particle size, which is generally the particle size of calcareous nodules with the largest content in samples. Second, there is a same interaction assumed between each contact of calcareous nodules. Then, steps to obtain contacts are as follows:

Step 1, the total volumes of calcareous nodules and fine soil in a sample with small size are calculated according to the CNC, as expressed by Eqs. (7) and (8). Then, the volume of the sample with small size can be expressed by Eq. (9).

7 $$V_{{\text{c,m,total}}} = \frac{m \cdot CNC}{{\rho_{{\text{c}}} }}$$

8 $$V_{{\text{s,m,total}}} = \frac{m \cdot (1 - CNC)}{{\rho_{{\text{s}}} }}$$

9 $$V_{{\text{m}}} = (V_{{\text{c,m,total}}} + V_{{\text{s,m,total}}} )(1 + e_{0} ) = \frac{{m(1 + e_{0} )[\rho_{{\text{s}}} \cdot CNC + \rho_{{\text{c}}} (1 - CNC)]}}{{\rho_{{\text{s}}} \rho_{{\text{c}}} }}$$

where $m$ represents the mass of the sample with a small size, $V_{{\text{c,m,total}}}$ represents the total volume of calcareous nodules in this sample with a small size, $CNC$ represents the content of calcareous nodule, $\rho_{{\text{c}}}$ represents the density of calcareous nodules, $V_{{\text{s,m,total}}}$ represents the total volume of fine soil in this sample with a small size, $\rho_{{\text{s}}}$ represents the density of fine soil, $V_{{\text{m}}}$ represents he volume of this sample with a small size, $e_{0}$ represents the initial void ratio of real samples.

Step 2, the volumes of a single calcareous nodule sphere and a real sample are obtained according to the calculation formula of the ball and cylinder, respectively, as expressed by Eqs. (10) and (11).

10 $$V_{{\text{c,single}}} = \frac{4}{3}\pi r^{3}$$

11 $$V = \pi R^{2} H$$

where $V_{{\text{c,single}}}$ represents the volume of a single calcareous nodule sphere, $r$ represents the radius of a single calcareous nodule sphere, $V$ represents the volume of a real sample, $R$ represents the radius of this real sample, $H$ represents the height of this real sample.

Step 3, the coefficient of sample reduction can be expressed by Eq. (12) according to Eqs. (9) and (11).

12 $$\xi = \frac{V}{{V_{{\text{m}}} }} = \frac{{\pi R^{2} H\rho_{{\text{s}}} \rho_{{\text{c}}} }}{{m(1 + e_{0} )[\rho_{{\text{s}}} \cdot CNC + \rho_{{\text{c}}} (1 - CNC)]}}$$

Step 4, the number of calcareous nodules in the sample according to the total volume of calcareous nodules [see Eq. (7)] and the volume of a single calcareous nodule sphere [see Eq. (10)] is calculated, as expressed by Eq. (13).

13 $$N_{{\text{m}}} = \frac{{V_{{\text{c,m,total}}} }}{{V_{{\text{c,single}}} }} = \frac{3}{4}\frac{m \cdot CNC}{{\pi r^{3} \rho_{{\text{c}}} }}$$

where $N_{{\text{m}}}$ represents the number of calcareous nodules with *r* in radius in the sample with a small size.

Step 5, contacts of calcareous nodules in the real sample is expressed by Eq. (14).

14 $$x = x_{{\text{m}}} \cdot \xi = \frac{{x_{{\text{m}}} \pi R^{2} H\rho_{{\text{s}}} \rho_{{\text{c}}} }}{{m(1 + e_{0} )[\rho_{{\text{s}}} \cdot CNC + \rho_{{\text{c}}} (1 - CNC)]}}$$

where $x$ represents contacts of calcareous nodules in the real sample, $x_{{\text{m}}}$ represents contacts of calcareous nodules in the sample with small size where $N_{{\text{m}}}$ calcareous nodules are evenly distributed, which can be obtained by counting.

**Figure 9:**
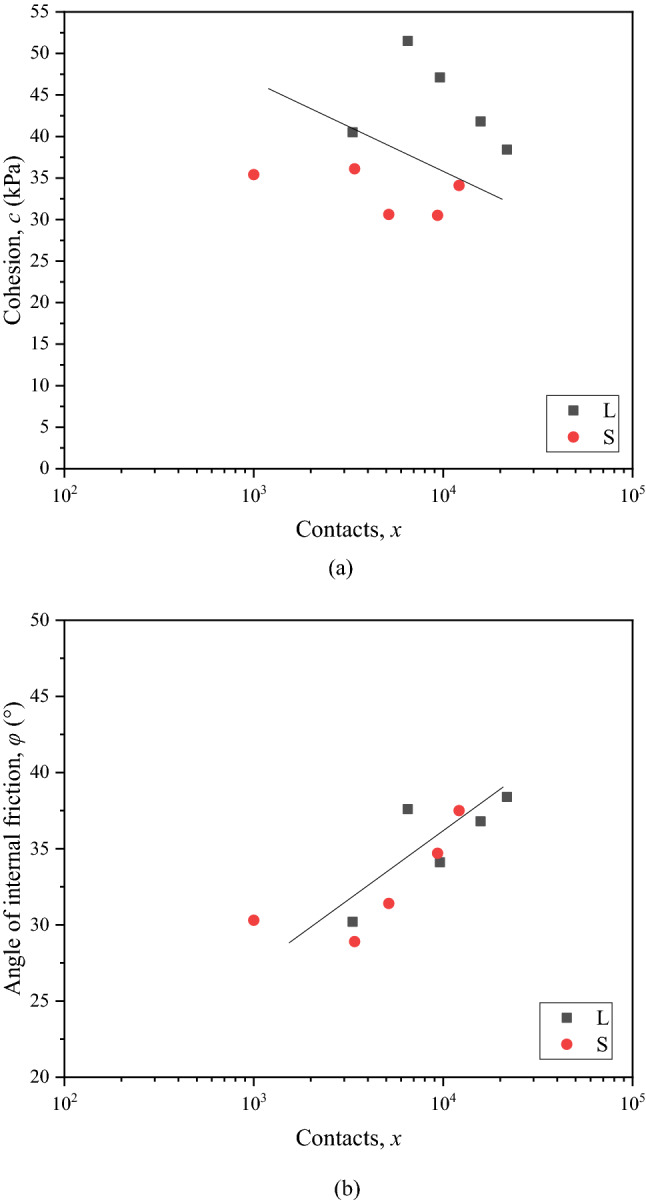
Relationship between (**a**) the cohesion, (**b**) internal friction angle and contacts of calcareous nodules.

Figure 9a and b show the relationship between the cohesion, internal friction angle and contacts obtained through above calculation steps, respectively, where L represents samples with large size and S represents samples with corresponding scaling size. Obviously, the increasing contacts results in a decreasing cohesion and an increasing internal friction angle. This is because that with the increase of contacts, the cementation of samples is weakened and the bite force of calcareous nodules is enhanced. This behavior is consistent with previous studies^24^ and suggests that the prediction results of contacts of calcareous nodules can be applied in the calculation model. It is worth noting that, the correlation between the cohesion, internal friction angle and contacts of CSCN samples can be expressed as a line, which can be expressed by Eqs. (15) and (16), respectively.

15 $$c = c_{0} + \lambda \cdot \lg x,\begin{array}{*{20}c} {} & {} \\ \end{array} x = 1,2, \cdot \cdot \cdot ,n.$$

16 $$\varphi = \varphi_{0} + \gamma \cdot \lg x,\begin{array}{*{20}c} {} & {} \\ \end{array} x = 1,2, \cdot \cdot \cdot ,n.$$

where: $c_{0}$ represents the cohesion of the sample at the CNC of 0%, $\lambda$ represents the tangent slope of the *c-x* curve, $\varphi_{0}$ represents the internal friction angle of the sample at the CNC of 0%, $\gamma$ represents the tangent slope of the $\varphi$*-x* curve.

**Figure 10:**
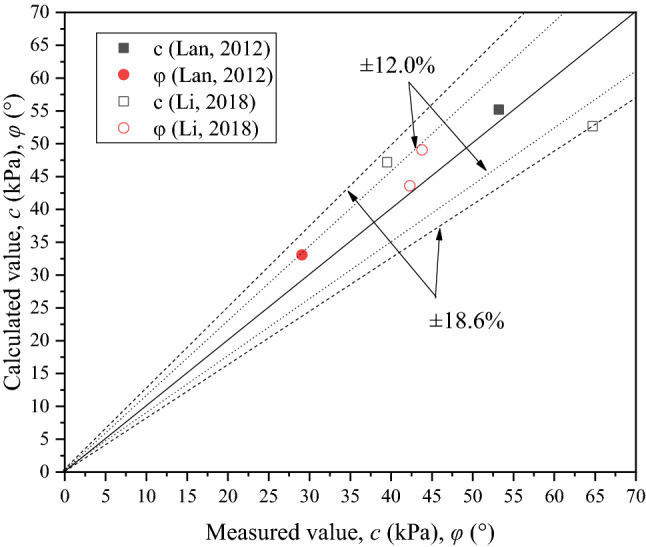
Comparison of calculated and measured cohesion and internal friction angle.

Some data taken form the published dissertations^32,33^ was used to validate the proposed calculation model. The degree of accuracy of the proposed calculation model was evaluated through a comparison of the calculated and measured values. Figure 10 shows the comparison of calculated and measured shear strength parameters. The difference of calculated and measured values is small and the largest difference is 12.04 kPa in the cohesion and 5.26° in the internal friction angle*.* The error bounds for the calculated cohesion and internal friction angle are ± 18.6% and ± 12.0%, respectively, which are lower than the average error (about 25%) evaluated using the Li’s method^6^. This suggests that calculated values are in a good agreement with measured values and it is reasonable to propose to adopt the calculation model for estimation of the shear properties of CSCN samples. It is also believed that the calculation model provides an access to obtain calculated shear strength parameters of CSCN samples with large size by using measured results of samples with corresponding scaling size.

## Conclusions

In this paper, direct shear tests were performed to investigate the influence of the CNC on the scaling effect in shear strength of CSCN samples. From the understanding of the reason for the influence, a calculation model for the shear strength parameters of CSCN samples was proposed. The conclusions are as follows.

The correlation between the scaling effect in shear strength and the CNC presents a step curve and the inflection point locates at the CNC of 20%–30%. This suggests that the scaling effect in the cohesion and internal friction angle is obvious for samples with 20%–30% in CNC while that is negligible for samples with a lower or higher CNC. Therefore, when the CNC is low (0%–10%) or high (more than 30%), it is applicable to adopt samples with scaling size to study the shear properties of the CSCN without considering the scaling effect.

The arrangement of scaling particles into downsized grain groups makes the soil structure be affected significantly by the particle size of calcareous nodules with the largest content. As the similar gradation composition, the ratio of the particle size of calcareous nodules with the largest content to the sample diameter for samples with corresponding scaling size is larger than that for samples with large size. This performance makes it is easier for calcareous nodules in samples with scaling size to form the skeleton structure to resist the compression stress during the consolidation, resulting in the skeleton effect working more easily to increase the scaling effect when shearing.

The calculation model proposed in this paper calculated the cohesion and internal friction angle of CSCN samples reasonably well. The error bound for the calculated cohesion and internal friction angle is approximately ± 18.6% and ± 12.0% of the measured values, respectively. It is believed that the calculation model outlined in this paper can provide an access to obtain calculated compression characteristic parameters of CSCN samples with large size by using measured results of samples with corresponding scaling size. Furthermore, the numerical analysis mixed with statistical analysis can be used as an attempt to simulate contacts of calcareous nodules in samples with different sizes in the further study, which can build a precise calculation model to provide guidance for construction.
